# Novel Biomaterial for Artery Patch in Swine Model With High-Fat Diet

**DOI:** 10.3389/fbioe.2021.679466

**Published:** 2021-06-17

**Authors:** Xiao Lu, Ling Han, Xiaomei Guo, Mengjun Wang, Sam Baradarian, Eugene Golts, Ghassan S. Kassab

**Affiliations:** ^1^California Medical Innovations Institute, San Diego, CA, United States; ^2^3DT Holdings, San Diego, CA, United States; ^3^Scripps Clinic Cardiovascular Surgery, San Diego, CA, United States; ^4^University of California San Diego (UCSD) Cardiovascular Surgery, San Diego, CA, United States

**Keywords:** pulmonary visceral pleura, patch-angioplasty, hypercholesterolemia, swine model, biocompatibility

## Abstract

**Objective:**

We evaluated swine and bovine pulmonary visceral pleura (PVP) in artery patch-angioplasty in swine model of high-fat diet.

**Background:**

Arterial patch-angioplasty is frequently used for repair or reconstruction of arteries. An autologous patch is often limited by the number and dimension of donor tissue and can result in donor complications. Furthermore, mechanical mismatch is a cause of poor performance of vascular reconstruction. Here, we introduce a readily available patch biomaterial with similar compliance as native arteries.

**Methods:**

The PVP was peeled from swine and bovine lungs by hydro-dissection. The swine and bovine PVPs were crosslinked with glutaraldehyde and then sterilized. The swine PVP (sPVP) patches were implanted in the carotid and femoral arteries of six Yorkshire pigs that were fed a regular diet and euthanized at 2 and 4 months postoperative. The bovine PVP (bPVP) patches were implanted in the carotid artery of six Yucatan pigs that were fed a high-fat diet and euthanized at 4 months postoperative. Patency was evaluated by ultrasound and angiography. Neo-endothelium and media were evaluated by histologic examination.

**Results:**

All arteries in patch-angioplasties remained patent with no adhesions, inflammation, or aneurysms. Biomarkers of endothelial cells (e.g., Factor VIII and eNOS) were detected in the neo-endothelial cells. We observed endothelial cell–cell junctions in the confluent neo-endothelium in the PVP patches. Neo-media composed of vascular smooth muscle developed similar as native arteries. In the hypercholesterolemic model, we observed the accumulation of cholesterol in both arterial tissues and in the neo-vascular tissues in the PVP patches. Protein expressions of lipid transport and metabolism (e.g., APOE-1, ABCA, and PACK9) were also observed in both arterial and neo-vascular tissues.

**Conclusion:**

The PVP patch-angioplasty overcomes the pitfalls of compliance mismatch of synthetic patches and has a non-thrombogenic surface. The proliferation of vascular cells assembled to generate the neo-endothelium and media in the patch-angioplasties to support long-term patency. The neo-vascular tissue in PVP patch-angioplasty also developed similar cellular functions for lipid transport and metabolism compared with native arteries in hypercholesterolemia.

## Introduction

Vascular patches (patch-angioplasty) are broadly used in blood vessel repair and reconstruction when blood vessels are severely compromised in cardiovascular diseases, infection, and/or transplantation (Muto et al., 2009; Harling et al., 2012; Hwang et al., 2012; Garcia Aroz et al., 2017). An autologous patch (mostly autologous vein harvesting) is generally the gold standard for arterial repair and reconstruction because there is no risk of inflammation and immune rejection (Muto et al., 2009). The availability of autologous patches is often limited by number and dimension of autologous veins, and even when available, the vein may lead to a compliance mismatch with the host arteries (O’Hara et al., 1992; Dardik et al., 1997). Synthetic biomaterials have been used as an alternative vascular patch source, although the rate of occlusion in synthetics is relatively high and development of infection may require life-long follow-up (Jeschke et al., 1999; Rizzo et al., 2000; Harling et al., 2012; Hwang et al., 2012). Xenogeneic patches such as bovine pericardium (Gonçalves et al., 2005; Maestro et al., 2006; Garcia Aroz et al., 2017) and porcine small intestinal submucosa (SIS) (Jernigan et al., 2004; Witt et al., 2013) are currently in development. The durability and outcomes of xenogeneic patches are comparable with native and synthetic patches, but lead to less suture line bleeding than synthetics and lower infection rates (Naylor et al., 2002; Smith et al., 2008; Muto et al., 2009; Kumar et al., 2013; Dimitrievska et al., 2015). The elasticity of the bovine pericardium and porcine SIS-ECM (extracellular matrix), however, is still not optimal for an artery tissue. It is known that mechanical mismatch is a cause of poor performance of vascular reconstruction (Zilla et al., 2007; Witt et al., 2013; Sánchez et al., 2018). The initial thrombogenicity of bovine pericardium and porcine SIS-ECM is also a serious concern for xenogeneic patch in vascular reconstruction (Witt et al., 2013; Sánchez et al., 2018). Thus, a vascular patch with better performances is needed in vascular surgery and has been a significant focus of research over several decades.

Here, we evaluated the swine and bovine pulmonary visceral pleura (PVP) as potential biomaterials for patch-angioplasty. The swine PVP (sPVP) patches were implanted in the carotid and femoral arteries of Yorkshire pigs to evaluate biocompatibility. The bovine PVP (bPVP) patches were implanted in the carotid artery of adult Yucatan pigs fed high-fat diet to mimic hypercholesterolemia while bovine pericardium (bPcdm) patches implanted in the lateral carotid artery served as controls (i.e., xenogeneic model). We assessed the patency and proliferation of the neo-vascular tissue in the patch-angioplasty for the both the sPVP and bPVP to measure the viable candidates for patching.

## Materials and Methods

### Preparation of Vascular Grafts

The swine and bovine lungs and bovine pericardium were obtained from a local abattoir and transported en bloc at 4°C to our facility within 4 h. The PVP was excised from the lungs. The PVP and bovine pericardium were fixed in 0.65% glutaraldehyde overnight and stored in 0.25% glutaraldehyde. A digital gauge (Model 547-500S; Mitutoyo) was used to measure the thickness of the sPVP, bPVP, and bPcdm. The thickness measurements were then averaged at five positions which resulted in an average sPVP thickness of ∼70 μm, an average bPVP thickness of ∼280 μm, and an average bPcdm thickness of ∼500 μm. All tissues were sterilized with a solution containing 2.05 g/L NaOH, 10.83 g/L PO_4_H_2_K, 200 ml/L alcohol, 40 ml/L 25% glutaraldehyde, and 110 ml/L 4% formaldehyde at 37°C for 24 h and then thoroughly rinsed in saline before implantation.

### Animal Experiments

Twelve Yorkshire pigs weighing 55 ± 7 kg (age 5 ± 1 months) and six Yucatan pigs weighing 62 ± 7 kg (age 6 ± 2 years) were obtained from a certified vendor. After acclimation, one sPVP patch was implanted in the common carotid artery of each Yorkshire pig and other sPVP patch in the common femoral artery of each Yorkshire pig. The pigs fed a regular diet were randomly assigned into the 2-Months Post-Op (2-M) Group (*n* = 6) and the 4-Months Post-Op (4-M) Group (*n* = 6). Six Yucatan pigs fed high-fat diet were assigned into the High Fat/4-Months Post-Op (HF/4-M) Group (*n* = 6). One bPVP patch and one bPcdm were implanted in the right and left common carotid arteries in every Yucatan pig. All animal experiments were performed in accordance with national and local ethical guidelines, including the Principles of Laboratory Animal Care, the Guide for the Care and Use of Laboratory Animals, and the National Society for Medical Research, as well as an approved California Medical Innovations Institute IACUC protocol regarding the use of animals in research.

The pigs were pre-anesthetized with Telazol (50 mg/ml), ketamine (25 mg/ml), and xylazine (25 mg/ml) and maintained with 2% isoflurane. The skin in the neck and groin was shaved and scrubbed for a sterile exposure of the arteries. After dissection, proximal and distal vascular clamps for hemostasis were applied to the arteries. The vascular wall of 8 × 20 mm and 6 × 18 mm (circumferential × axial) was excised from carotid and femoral artery arteries, respectively. The sPVP, bPVP, or bPcdm patches were trimmed to the size of vascular excisions. The patches were sutured on the vascular excisions with the aid of a surgical microscope. Blood flow was re-established with removal of the clamps. The incisions were closed in two layers. The animals were allowed free access to food and water. Animals were given Buprenorphine, Carprofen, Excede, and Baytril for postoperative analgesia and infection control for 3 days. Clopidogrel (75 mg/day) and aspirin (325 mg/day) were administered orally for survival durations. Patency of the patched blood vessels was assessed once every 4 weeks by ultrasound and angiography at terminal study.

In the terminal studies, the animals were anesthetized and heparinized. Catheterization was established for angiography as per standard interventional preparation. Digital model images (Veradius Unity; Philips) were recorded during contrast infusion in the patched arteries. After the angiography procedures, neck and inguinofemoral incisions were made to expose the carotid artery and femoral artery implanted patches, respectively. Visual assessment of potential aneurysm or inflammation was made. After euthanasia, the patches were explanted with an additional ∼1 cm artery segment at both ends.

### Histology and Immunofluorescence Microscopy

The tissue segments were fixed with 4% paraformaldehyde at physiologic pressure. The middle portions of the patches, as well as segments of the proximal and distal arteries, were sampled. Transverse sections of the samples were cut using a cryotome (CM1850; Leica). The sections were stained with H&E. The cross-section area (CSA) of the patches and arteries was measured. The luminal ratio was represented by the ratio of patch CSA to artery CSA. For immunofluorescence, sections were rinsed three times in phosphate buffer solution (PBS, pH 7.4). The tissues were blocked in 3% bovine serum albumin for 30 min at 22°C and washed three times in PBS. The sections were incubated with the primary antibodies, including smooth muscle cell (SMC) biomarker: anti-SMC α-actin; endothelial cell (EC) biomarkers: anti-Factor VIII and anti-eNOS (endothelial nitric oxide synthase); and cholesterol-related biomarkers: Filipin (fluorescence dye of cholesterol) (Maxfield and Wüstner, 2012; Wilhelm et al., 2019), anti-7-keto cholesterol, anti-ABCA (ATP-binding cassette A), anti-PCSK9 (proprotein convertase subtilisin/kexin type 9), and anti-ApoE (apolipoprotein E). After the PBS rinse, the sections were incubated with fluorescent secondary antibodies. The images were obtained using a fluorescence microscope (Eclipse Ts2R; Nikon).

To visualize the endothelial cell lining on the neo-vascular tissue, we prepared ∼2 × 2-mm pieces of neo-vascular tissue for en face image of immunofluorescence microscopy. The pieces of tissue were excised from the neo-vascular tissues (implanted patches) and the native arterial tissues (control) and trimmed to a thin sheet. The thin sheets were incubated with the primary antibodies to represent endothelial cell–cell adherens junctions: anti-VE cadherin and anti-PECAM-1 (platelet endothelial cell adhesion molecule). The thin sheets were then incubated with fluorescent secondary antibodies and flattened on cover slides. The endothelium was faced to the objective of fluorescence microscope. The endothelial cell–cell junctions were visualized using a fluorescence microscope (Eclipse Ts2R; Nikon).

### Orientation Analysis

We measured the length to width aspect ratio of vascular SMC to estimate the orientation of these cells in the neo region between the patch and lumen (Chen et al., 2013). In the fluorescence images for visualized α-actin, both width and length of SMCs were measured. The aspect ratio was computed for each cell. The probability density function of the aspect ratio was analyzed.

### Statistical Analysis

The data were presented as mean ± SD and significant differences between two groups were determined by Student’s *t*-test (two-tailed distribution, two-sample unequal variance). A probability of *p* < 0.05 was indicative of a statistically significant difference.

## Results

**FIGURE 1:**
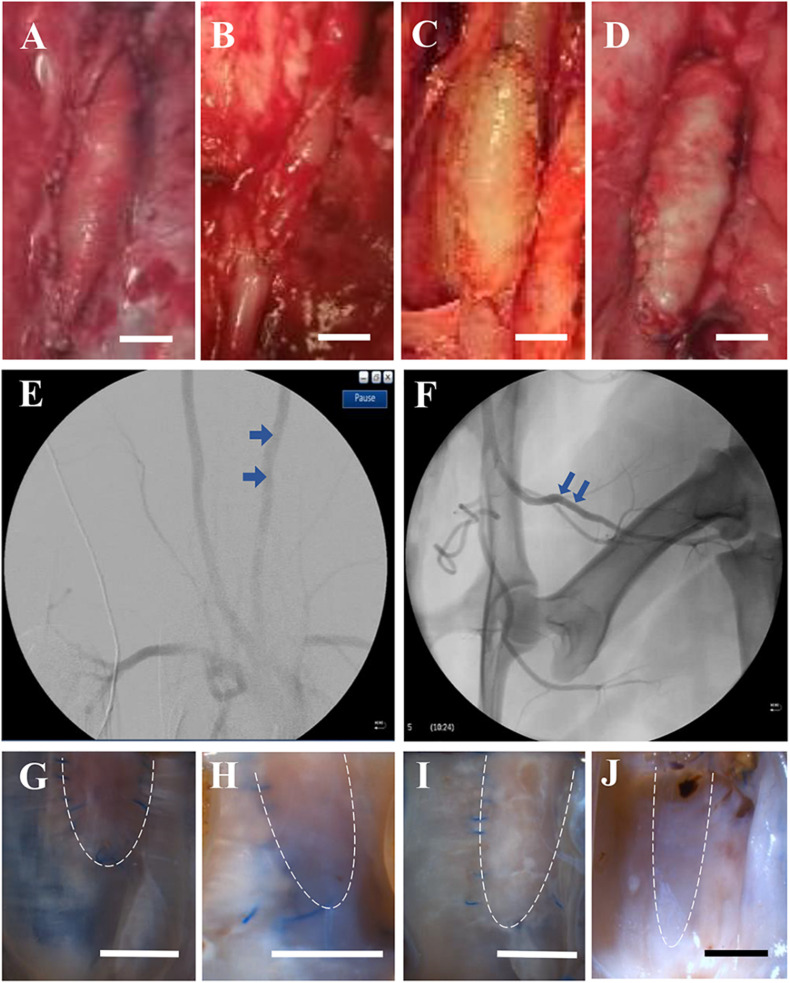
Typical gross and angiograph images of patch-angioplasties. **(A)** The sPVP in the carotid artery in 4-M Group. **(B)** The sPVP in the femoral artery in 4-M Group. **(C)** The bPVP in the carotid artery in HF/4-M Group. **(D)** The bovine pericardium in the carotid artery in HF/4-M Group. **(E)** The angiographic image of the bPVP in the carotid artery in HF/4-M Group. **(F)** The angiographic image of the sPVP in the femoral artery in 4-M Group. **(G)** The luminal surface of the sPVP in the carotid artery in 4-M Group. **(H)** The luminal surface of the sPVP in the femoral artery in 4-M Group. **(I)** The luminal surface of the bPVP in the carotid artery in HF/4-M Group. **(J)** The luminal surface of the bPcdm in the carotid artery in HF/4-M Group. The bars on **(A–D,G–J)** are the scale of 5 mm. The dash lines on **(G–J)** indicate the fractional patch-angioplasties (other tissues were processed for histology/immunofluorescence).

**FIGURE 2:**
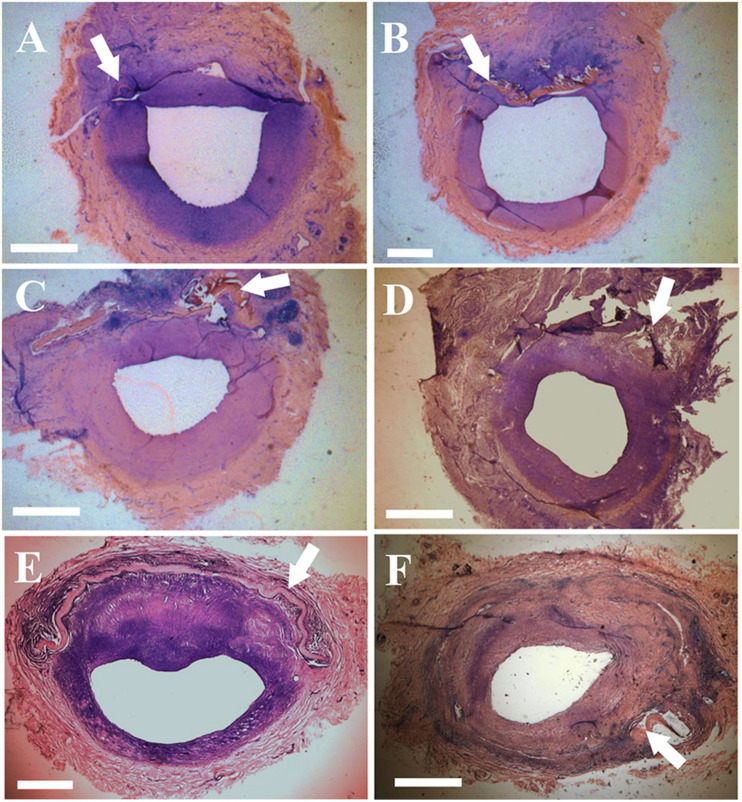
Histological images (H&E stain) of the patch-angioplasties. **(A)** The sPVP in the carotid artery in 2-M Group. **(B)** The sPVP in the carotid artery in 4-M Group. **(C)** The sPVP in the femoral artery in 2-M Group. **(D)** The sPVP in the femoral artery in 4-M Group. **(E)** The bPVP in the carotid artery in HF/4-M Group. **(F)** The bovine pericardium in the carotid artery in HF/4-M Group. White arrows indicate patches. Bar: 1 mm.

Figures 1A–D shows examples of patches that were sutured in the carotid and femoral arteries. Ultrasound and angiography in post-op examination confirmed the patency in the patch-angioplasties. The typical angiograph images are represented in Figures 1E,F. In the terminal studies, no blockages, aneurysms, adhesions, or inflammation were observed in any patch-angioplasties. The patches were integrated on the native arteries. Typical examples of the luminal surface in the patch-angioplasties are represented in Figures 1G–J. No calcification was observed in the patch-angioplasties (i.e., negative Alizarin Red staining). The H&E stains are represented in Figure 2. The luminal ratios of the PVP patch-angioplasties to native segments were 0.96 ± 0.04 and 0.98 ± 0.05 for carotid and femoral patch-angioplasties in 4-M Group, respectively, which did not significantly differ from the luminal ratios of 0.93 ± 0.05 and 0.94 ± 0.06 for the carotid and femoral patch angioplasties in 2-M Group 1 (*p* > 0.05) which indicates non-stenosis. Alternatively, the luminal ratio of the bPcdm patch-angioplasties to the native segments is 0.62 ± 0.27, which is significantly smaller than that (0.97 ± 0.06) of the bPVP patch-angioplasties (*p* < 0.05) in HF/4-M Group which suggests stenosis in the bPcdm patches.

**FIGURE 3:**
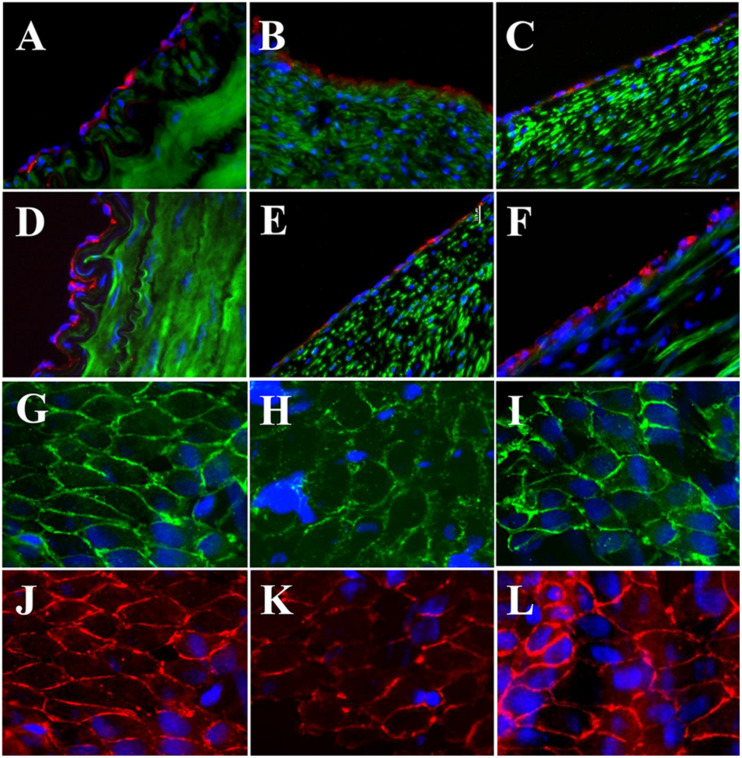
Expression of endothelial biomarkers in neo-endothelial cells of carotid patch-angioplasty in 2-M Group and in 4-M Group. **(A–F)** Transverse. **(A)** Factor VIII in native artery. **(B)** Factor VIII in 2-M Group. **(C)** Factor VIII in 4-M Group. **(D)** eNOS in native artery. **(E)** eNOS in 2-M Group. **(F)** eNOS in 4-M Group. **(G–L)**: En face. **(G)** VE cadherin in native artery. **(H)** VE cadherin in 2-M Group. **(I)** VE cadherin in 4-M Group. **(J)** PECAM-1 in native artery. **(K)** PECAM-1 in 2-M Group. **(L)** VE cadherin in 4-M Group. Red in **(A–C)** anti-Factor VIII. Red in **(D–F)** anti-eNOS. Green in **(A–F)** anti-SMC α-actin. Green in **(G–I)** anti-VE cadherin. Red in **(J–L)** anti-PECAM-1. Blue in all: nuclei of cells. Objective: 60×.

The expressions of endothelial cell (EC) biomarkers in carotid arteries and sPVP patch-angioplasties in 2-M and 4-M Groups are represented in Figure 3. The expressions of Factor VIII in the native EC, neo-EC in 2-M Group, and neo-EC in 4-M Group are shown (transverse views) in Figures 3A–C, respectively. The expressions of eNOS in the native EC, neo-EC in 2-M Group, and neo-EC in 4-M Group are similarly represented in Figures 3D–F, respectively. The expressions of VE cadherin in the cell–cell adherens junctions of the native EC, neo-EC in 2-M Group, and neo-EC in 4-M Group are shown in the en face images in Figures 3G–I, respectively. The expression of PECAM-1 in the cell–cell adherens junctions of the native EC, neo-EC in 2-M Group, and neo-EC in 4-M Group can be seen in the en face images in Figures 3J–L, respectively.

**FIGURE 4:**
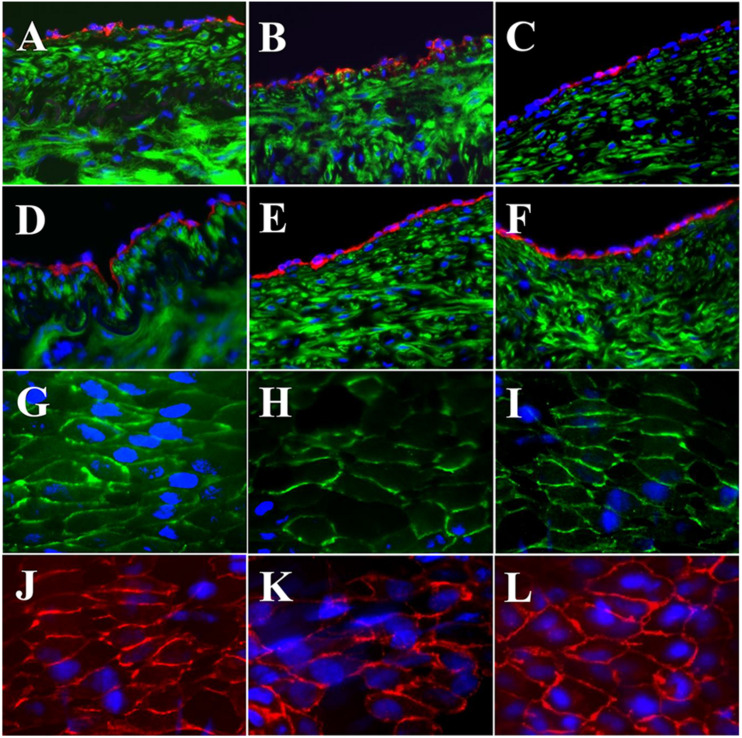
Expression of endothelial biomarkers in neo-endothelial cells of femoral patch-angioplasty in 2-M and 4-M Groups. **(A–F)** Transverse. **(A)** Factor VIII in native artery. **(B)** Factor VIII in 2-M Group. **(C)** Factor VIII in 4-M Group. **(D)** eNOS in native artery. **(E)** eNOS in 2-M Group. **(F)** eNOS in 4-M Group. **(G–L)** En face. **(G)** VE cadherin in native artery. **(H)** VE cadherin in 2-M Group. **(I)** VE cadherin in 4-M Group. **(J)** PECAM-1 in native artery. **(K)** PECAM-1 in 2-M Group. **(L)** VE cadherin in 4-M Group. Red in **(A–C)** anti-Factor VIII. Red in **(D–F)** anti-eNOS. Green in **(A–F)** anti-SMC α-actin. Green in **(G–I)** anti-VE cadherin. Red in **(J–L)** anti-PECAM-1. Blue in all: nuclei of cells. Objective: 60×.

The expressions of EC biomarkers in femoral arteries and sPVP patch-angioplasties in 2-M Group and 4-M Group are shown in Figure 4. The expression of Factor VIII in the native EC, neo-EC in 2-M Group, and neo-EC in 4-M Group are represented (transverse views) in Figures 4A–C, respectively. The expression of eNOS in the native EC, neo-EC in 2-M Group, and neo-EC in 4-M Group can be seen in Figures 4D–F, respectively. The expressions of VE cadherin in the cell–cell adherens junctions of the native EC, neo-EC in 2-M Group, and neo-EC in 4-M Group are shown in Figures 4G–I, respectively. Finally, the expression of PECAM-1 in the cell–cell adherens junctions of the native EC, neo-EC in 2-M Group, and neo-EC in 4-M Group are shown in Figures 4J–L, respectively.

**FIGURE 5:**
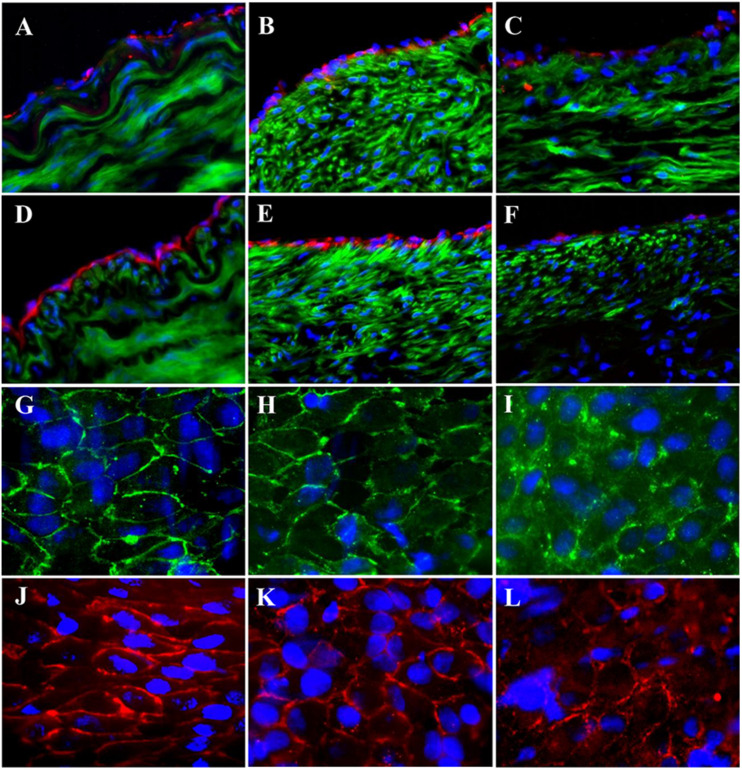
Expression of endothelial biomarkers in neo-endothelial cells of carotid patch-angioplasty in HF/4-M Group. **(A–F)** Transverse. **(A)** Factor VIII in native artery. **(B)** Factor VIII in bPVP patch. **(C)** Factor VIII in bPcdm patch. **(D)** eNOS in native artery. **(E)** eNOS in bPVP patch. **(F)** eNOS in bPcdm patch. **(G–L)** En face. **(G)** VE cadherin in native artery. **(H)** VE cadherin in bPVP patch. **(I)** VE cadherin in bPcdm patch. **(J)** PECAM-1 in native artery. **(K)** PECAM-1 in bPVP patch. **(L)** PECAM-1 in bPcdm patch. Red in **(A–C)** anti-Factor VIII. Red in **(D–F)** anti-eNOS. Green in **(A–F)** anti-SMC α-actin. Green in **(G–I)** anti-VE cadherin. Red in **(J–L)** anti-PECAM-1. Blue in all: nuclei of cells. Objective: 60×.

High-fat diet for the pigs in HF/4-M Group significantly increased the swine lipid level. Triglyceride (mg/dl) and cholesterol (mg/dl) increased from 30.8 ± 7.1 and 67.0 ± 7.3 to 59.7 ± 21.6 (*p* < 0.05) and 618.8 ± 75.9 (*p* < 0.01), respectively. HDL and LDL cholesterol (mg/dl) increased from 32.6 ± 5.4 and 33.4 ± 4.5 to 102.3 ± 11.2 (*p* < 0.01) and 237.4 ± 66.3 (*p* < 0.01). The expressions of EC biomarkers in the carotid artery, bPVP, and bPcdm patch-angioplasties in HF/4-M Group are shown in Figure 5. The expression of Factor VIII in the native EC, neo-EC in the bPVP, and neo-EC in the bPcdm in HF/4-M Group can be seen in the images of the transverse view in Figures 5A–C, respectively. The expressions of eNOS in the native EC, neo-EC in the bPVP, and neo-EC in the bPcdm in HF/4-M Group are represented in the images of the transverse view in Figures 5D–F, respectively. The expression of VE cadherin in the cell–cell adherens junctions of the native EC, neo-EC in the bPVP, and neo-EC in the bPcdm in HF/4-M Group can be seen in the en face images in Figures 5G–I, respectively. The expressions of PECAM-1 in the cell–cell adherens junctions of the native EC, neo-EC in the bPVP, and neo-EC in the bPcdm in HF/4-M Group are represented in the en face images in Figures 5J–L, respectively.

**FIGURE 6:**
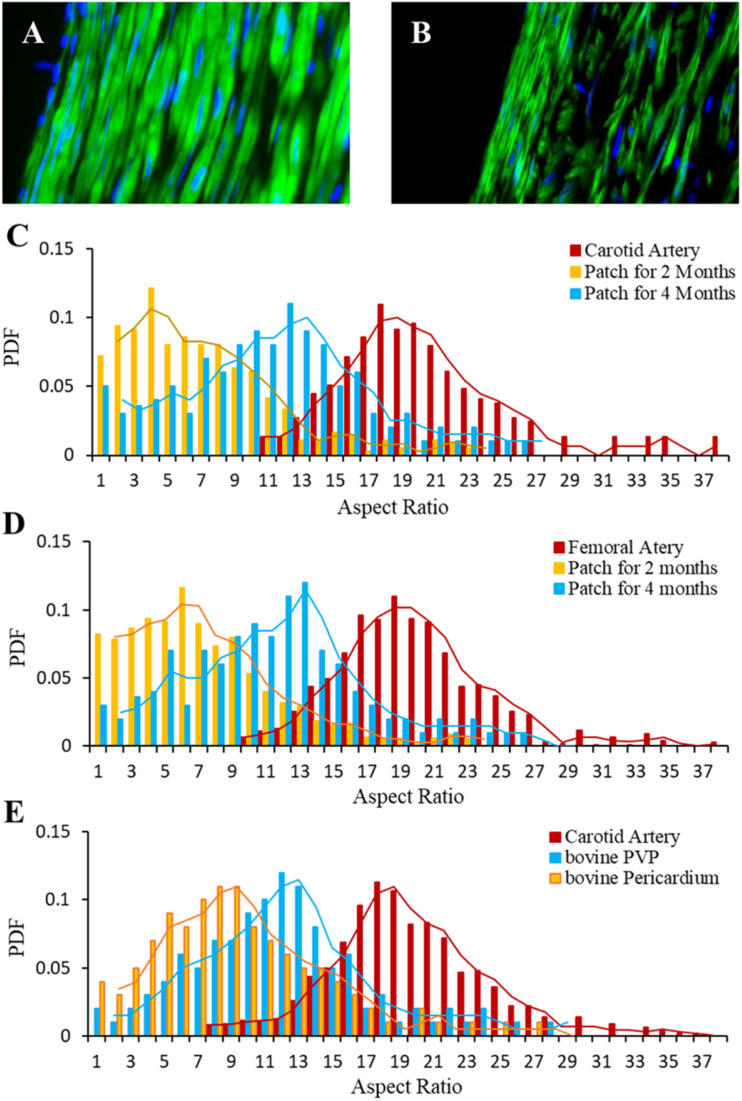
Orientation of myofibroblasts in neo-media of patch-angioplasties. **(A)** Carotid media. **(B)** Neo-media of sPVP in 4-M Group. Green: anti-smooth muscle α-actin. Blue: nuclei. Objective: 60×. **(C)** PDF vs. aspect ratios of the sPVP in carotid artery. **(D)** PDF vs. aspect ratios of the sPVP in femoral artery. **(E)** PDF vs. aspect ratios of the bPVP and pericardium in carotid artery in HF/4-M Group.

The immunofluorescence of smooth muscle α-actin antibody represents SMCs/myofibroblasts in carotid media (Figure 6A) and the neo-media (Figure 6B) suggesting SMC proliferation in the patches. The probability density function (PDF) was analyzed and the distribution of the PDF versus aspect ratio is shown in Figures 6C–E. Because the vessel segments were sectioned transversely, the aspect ratio approximately represents the orientation of neo-smooth muscle cells. The peaks of PDF are approximately 18 and 17 for carotid and femoral media, respectively. The peaks of PDF in the regions of patch-angioplasties were more scattered at 2-month than 4-month post-op (Figures 6C,D) in the Yorkshire pigs. The peaks of the PDF in the regions of all vessels at the 4-month post-op shifted toward those in native medial layers (Figures 6C–E).

**FIGURE 7:**
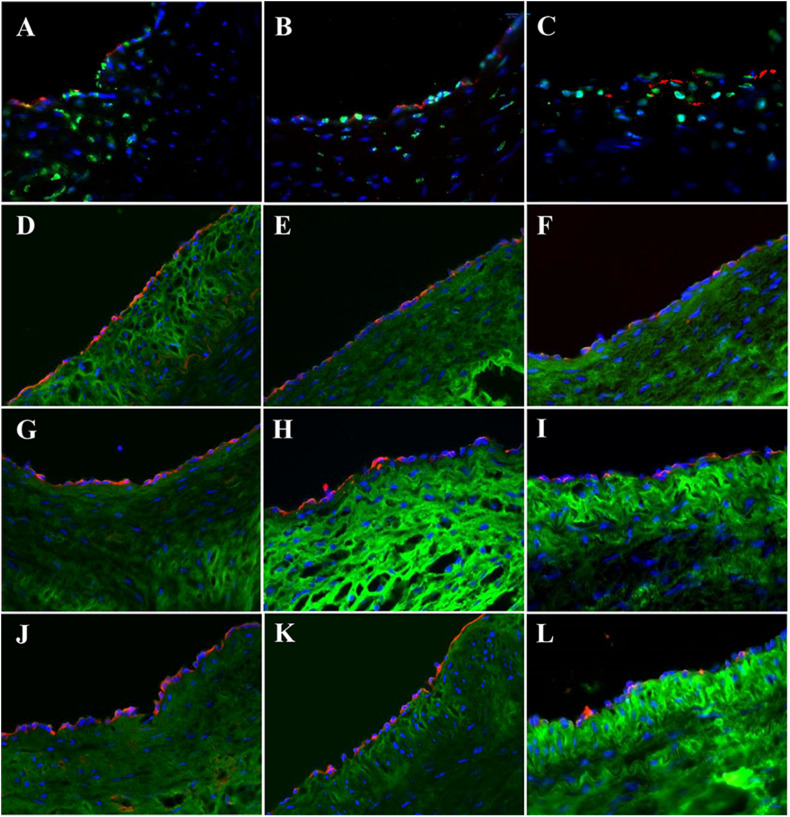
Cholesterol accumulation, transport, and metabolism in carotid patch-angioplasty in HF/4-M Group. **(A)** Filipin and 7-keto cholesterol in native artery. **(B)** Filipin and 7-keto cholesterol in bPVP patch. **(C)** Filipin and 7-keto cholesterol in bPcdm patch. **(D)** ABCA in native artery. **(E)** ABCA in bPVP patch. **(F)** ABCA in bPcdm patch. **(G)** PSCK in native artery. **(H)** PSCK in bPVP patch. **(I)** PSCK in bPcdm patch. **(J)** ApoE-1 in native artery. **(K)** ApoE-1 in bPVP patch. **(L)** ApoE-1 in bPcdm patch. Green in **(A–C)** Filipin. Red in **(A–C)** anti-7-keto cholesterol. Green in **(D–L)** anti-sialic acid (wheat germ agglutinin). Red in **(D–F)** anti-ABCA. Red in **(G–I)** anti-PSCA9. Red in **(J–L)** anti-ApoE-1. Blue in all: nuclei of cells. Objective: 60×.

The lipid and lipoprotein biomarkers were observed in HF/4-M Group. Filipin, a fluorescence dye, was used to detect the cholesterol in native arterial tissue and neo-vascular tissue of the patch (Maxfield and Wüstner, 2012; Wilhelm et al., 2019). Filipin positive was detected in both arterial and neo-vascular tissues in patch-angioplasties (Figures 7A–C, green). The 7-keto cholesterol, a typical biomarker of atherosclerosis, was also detected in both arterial and neo-vascular tissues in patch-angioplasties (Figures 7A–C, red). The ABCA (a lipo-metabolism–related protein) (Pinto et al., 2018) was expressed not only in arterial tissue but also in the neo-vascular tissue in patch-angioplasties (Figures 7D–F). PACK9 (a lipo-transportation–related protein) (Elseweidy et al., 2019) was expressed in arterial tissue and the expression in neo-vascular tissue in patch-angioplasties was also robust (Figures 7G–I). The ApoE-1 was expressed in arterial tissue and in neo-vascular tissue of patch-angioplasties (Figures 7J–L).

## Discussion

We used PVP in patch-angioplasties to repair common carotid and femoral arteries in normal and hypercholesterolemic pigs. The arteries in patch-angioplasties were found to be patent in the 4-month period in all animals. No stenosis or calcification was detected in the arteries with the PVP patch-angioplasties. Immuno-histological analysis demonstrated that neo-endothelial cells lined on the luminal surface of the PVP patch-angioplasties and neo-media developed in the PVP patch-angioplasties. The neo-vascular tissue in the PVP patch-angioplasties had similar characteristics to arterial tissue in hypercholesterolemia. The current study strongly supports PVP as a viable candidate for patch-angioplasties for vascular repair/reconstruction.

Bovine and swine PVP contain abundant elastin and the ratio of elastin to collagen is approximately 1:1 which is significantly different from existing biomaterials based on ECM (Lu et al., 2019), such as pericardium, peritoneum, and SIS which have a collagen/elastin ratio > 40:0 (Zilla et al., 2007). Because elastin and collagen are the major components of ECM, the PVP is undoubtedly of low cytotoxicity and has excellent biocompatibility (Lu et al., 2019). Abundant elastin renders the elasticity of the PVP close to arterial tissue, which minimizes the compliance mismatch between the patch and artery (Lu et al., 2019). In addition to the elasticity match, PVP has a reduced risk of thrombosis because elastin does not contain the RGD sequence (Arg-Gly-Asp) which is known to interact with integrins expressed on platelets and lead to platelet activation (Almine et al., 2010). Other advantages of using PVP as a vascular patch include that elastin is known as a potent autocrine regulator of vascular smooth muscle cell activity and inducer of actin stress fiber organization. Elastin also regulates myofibroblast activity and promotes quiescent fibroblasts (convert from genotype to phenotype state) (Rodgers and Weiss, 2005; Almine et al., 2010). The present study of the luminal ratio of the PVP-angioplasties implicates the utility of elastin in the PVP patch-angioplasty. The luminal ratio of the PVP patch-angioplasties in all groups (0.96–0.98) indicates no stenosis whereas the luminal ratio of the (0.62) indicates stenosis in the bPcdm patch-angioplasties.

The expressions of Factor VIII, eNOS, PECAM-1, and VE cadherin in the study suggest the successful neo-reendothelialization of the PVP patch angioplasties (Figures 3–5). We identified that endothelial cell–cell junctions (VE cadherin and PECAM-1 are endothelial adhesion molecules of adherens junctions) robustly developed in the neo-endothelium to render a confluent layer on the PVP which is important in endothelial barrier function and critical for thrombosis resistance of the PVP patch in long-term patency (Figures 3, 5G–L). The abundant smooth muscle α-actin between the lumen and the PVP patch indicates neo-media formation (Figure 6).

We measured the length and width of vascular smooth muscle cells (VSMCs) in the neo-media and analyzed the distribution of length/width ratio to determine the orientation of VSMCs. We previously reported that VSMCs circumferentially orientate in coronary media (Chen et al., 2013). In the early stage of neo-medial formation, synthetic VSMCs and myofibroblasts migrate into the lesion and orient randomly. Over time, synthetic VSMCs and myofibroblasts can develop contractile phenotype with abundant smooth muscle α-actin and myosin. Exposed to hemodynamic stimulation, the VSMCs gradually orient in the circumferential direction. Therefore, VSMC orientation is a morphometric indicator of vascular medial maturity in the neo-medial formation. Our observation shows that the distribution of VSMCs orientation in the neo-media in the PVP patch-angioplasties shifts toward the direction of the native media. The PVP patch is replaced with functional endothelium and mature neo-media to become native-like vascular tissue.

In this study, we investigated the PVP patches in pigs of two different ages. The Yorkshire pigs were ∼5 months old and Yucatan mini pigs were ∼6 years old. It is known that regeneration is robust in young animals and is relatively compromised in aging. Therefore, investigating the PVP patches in swine models of different ages is informative for clinical translation. However, there was no difference in the vascular proliferation in the PVP patch-angioplasty between two ages, which suggests that the PVP patch can be used in a broad range of ages.

In addition, hypercholesterolemia is one of the risk factors in cardiovascular diseases. Hypercholesterolemia results in vascular dysfunction, including endothelial dysfunction and hyperplasia, stenosis, and vascular wall stiffening (increase in fibrosis), and can ultimately progress to atherosclerosis. Unfortunately, vascular repair/reconstruction is often required in patients with cardiovascular diseases including hypercholesterolemia. Therefore, it was necessary to evaluate the safety and efficacy of the PVP in patch-angioplasties in a hypercholesterolemic model, for which we used an established hypercholesterolemic model used in biomedical research, e.g., Yucatan mini pigs fed with high-fat diet (Reitman et al., 1982). In the hypercholesterolemic model, we observed accumulation of cholesterol in arterial tissues which is consistent with previous studies (Figure 7) (Reitman et al., 1982; Zhao et al., 2018). A similar accumulation of cholesterol was also observed in neo-vascular tissues in the PVP patch-angioplasties. This suggests that both arterial and neo-vascular tissues in the PVP respond similarly in hypercholesterolemia. The protein expression of lipid transport and metabolism (e.g., APOE-1, ABCA, and PACK9) was observed in arterial and neo-vascular tissues (Figure 7). This suggests that the neo-vascular tissue exhibits similar cellular behavior for lipid transport and metabolism compared with the native artery in pigs (Zhao et al., 2018).

The cardiovascular system in Yorkshire pigs is similar to humans in dimension and structure and therefore is a good translational model for the evaluation of safety and potential efficacy of the PVP vascular patch. However, when the Yorkshire pig body weight is similar to adult patients (∼70 kg), the Yorkshire pig is pre-mature and the cardiovascular system has a higher regeneration capacity. When it is mature, the Yorkshire swine body weight is > 200 kg which is much larger than the average patient. Retired Yucatan breeder minipigs have a comparable body weight to humans (∼60 kg) which makes them an ideal hypercholesteremic model although the dimensions of the blood vessels are smaller than adult humans. Unfortunately, there is limited availability of these retired Yucatan breeder minipigs and the cost is high. Therefore, we used Yorkshire swine in evaluation of safety and performance in hyperlipidemic model (Yucatan pigs).

## Conclusion

In conclusion, the bovine and porcine PVP have thrombosis-resistant surfaces in patch-angioplasties that maintain patency and serve to host native vascular cells to proliferate neo-vascular tissue. The PVP used in patch-angioplasties may overcome the pitfalls of the compliance mismatch between synthetic patches and native blood vessels given the artery-like mechanical properties of the PVP patch. The neo-vascular tissue in PVP patch-angioplasties exhibits similar cellular behavior for lipid transport and metabolism to native artery in hypercholesterolemia. The neo-endothelium and neo-media formation observed in the two swine models support the long-term patency and warrant a first-in-human investigation.

## Data Availability Statement

The raw data supporting the conclusions of this article will be made available by the authors, without undue reservation.

## Ethics Statement

The animal study was reviewed and approved by California Medical Innovations Institute IACUC.

## Author Contributions

XL: contributions in conception, design, acquisition and analysis data, drafting article, and final approval. LH: contributions in acquisition and analysis, drafting article, and final approval. XG and MW: contributions in acquisition, drafting article, and final approval. SB and EG: contributions in design, interpretation of data, revising article, and final approval. GK: contributions in concept, design, interpretation of data, drafting and revising article, and final approval. All authors contributed to the article and approved the submitted version.

## Conflict of Interest

GK was the founder of 3DT Holdings. The remaining authors declare that the research was conducted in the absence of any commercial or financial relationships that could be construed as a potential conflict of interest.
